# Molecular Pathways Modulated by Curcumin Analogue, Diarylpentanoids in Cancer

**DOI:** 10.3390/biom9070270

**Published:** 2019-07-10

**Authors:** Felicia Paulraj, Faridah Abas, Nordin H. Lajis, Iekhsan Othman, Rakesh Naidu

**Affiliations:** 1 Jeffrey Cheah School of Medicine & Health Sciences, Monash University Malaysia, Jalan Lagoon Selatan, Bandar Sunway 47500, Selangor, Malaysia; 2 Laboratory of Natural Products, Faculty of Science, Universiti Putra Malaysia, UPM Serdang 43400, Selangor, Malaysia; 3 Department of Food Science, Faculty of Food Science and Technology, Universiti Putra Malaysia, UPM Serdang 43400, Selangor, Malaysia

**Keywords:** diarylpentanoid, curcumin, anticancer activity, molecular pathway, cancer

## Abstract

While curcumin has a range of therapeutic benefits, its potent anticancer activity remains an attractive avenue for anticancer research owing to the multifactorial nature of cancer itself. The structure of curcumin has thus been used as a lead to design more potent analogues, and diarylpentanoids in particular have shown improved cytotoxicity over curcumin. Investigations of diarylpentanoids have demonstrated that these compounds exert anti-cancer effects through several signalling pathways that are associated with cancer. This review focuses on selected diarylpentanoids and highlights molecular targets that modulate key pathways involved in cancer such as NF-κB, MAPK/ERK, and STAT signalling. Future research will need to focus on drug interactions to explore potential synergistic actions of diarylpentanoids and further establish the use of diverse animal models.

## 1. Introduction

The undesirable side effects associated with chemotherapeutics have prompted research into a range of other alternatives, including dietary phytochemicals that demonstrate anticancer effects. Natural agents often have lower toxicity and target multiple signalling pathways, making them efficacious in the treatment of cancers [1].

Turmeric is derived from *Curcuma longa* rhizhomes of the Zingiberaceae family and is native to South East Asia. The perennial herb is known as ‘The Golden Spice of India’ due to its yellow colour and its use as a key ingredient in curry powder, mainly in Indian cuisine [2]. In addition to its use in food, turmeric has been used in traditional medicine for more than 40 centuries as an herbal remedy for conditions such as inflammation of the liver, stomach, and kidney, to treat colic, for the treatment of scars and fevers, and in skin ailments [2]. Various preparations of turmeric extracts have been observed to produce a wide range of in vitro anti-oxidative, anti-tumorigenic, anti-inflammatory, and anti-microbial effects (as reviewed [3]). Turmeric contains a wide range of phytochemicals, amongst which curcumin has been identified as the active ingredient for the therapeutic effects of turmeric.

**Figure 1:**
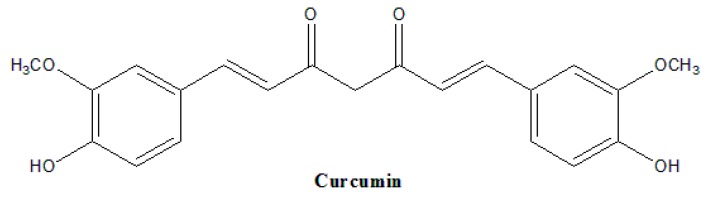
Molecular structure of curcumin.

Chemically known as diferuloylmethane (bis-α,β-unsaturated β-diketone), curcumin consists of about 3% to 5% turmeric. Curcumin (Figure 1) possesses two aromatic rings connected by a seven carbon tether that contains two α,β-unsaturated ketone moieties, which is enolizable depending on its aqueous surroundings [4]. The diketone moieties act as good Michael acceptors, and may undergo a nucleophilic attack when interacting with other molecular targets [5]. Curcumin is hydrophobic by nature and solubilises poorly in water but dissolves completely in most organic solvents [4].

Curcumin exhibits an exhaustive range of anti-inflammatory, anti-oxidative, anti-tumour, hypoglycaemic, hepatoprotective, anti-lipoperoxidation, and anti-platelet effects [6,7,8]. The anti-inflammatory effects of curcumin were mostly mediated through the regulation of the pro-inflammatory transcription factor, NF-κB. Curcumin has been shown to suppress the NF-κB pathway, specifically by inhibiting the activity of IκB kinase signalling [9]. Other inflammatory mediators affected by curcumin include the inhibition of signal transducers and activators of transcription (specifically STAT-3) expression, the inhibition of cyclooxygenase-2 (COX-2) [10], tumor necrosis factor alpha (TNFα), and interleukins [11]. In addition, curcumin possesses anti-oxidative properties that are suggested to contribute to its anti-inflammatory abilities. Studies report that treatment with curcumin increases reactive oxidative species (ROS) and glutathione production and inhibits lipid peroxidation [12].

In recent years, curcumin has been widely studied as a potential candidate for chemotherapy. Not only does curcumin have anti-metastatic and anti-proliferative effects on cancer cell growth, it also interacts with and regulates a diverse array of transcription factors, inflammatory cytokines, kinases, enzymes, and growth factors [5,13], which are critical intracellular components involved in cancer. Curcumin is associated with the activation of caspases; pro-apoptotic and tumour suppressor genes; the downregulation of matrix metalloproteinases (MMPs); the suppression vascular endothelial growth factor (VEGF); and many other factors that are involved in key processes such as genomic modulations, cell invasion, and cell death pathways [14,15,16]. Curcumin has been shown to interact with numerous other molecular targets and signalling pathways, including nuclear factor erythroid 2-related factor (Nrf-2), CREB-binding protein, epidermal growth factor (EGF), aryl hydrocarbon receptor (AHR), transforming growth factor 1 (TGF-1), death receptors (DR), heat shock protein 70 (Hsp70), protein kinases (PKA, PKB, and PKC), c-jun N-terminal kinase (JNK), intracellular adhesion molecule-1 (ICAM1), and NF-κB, among others [12,17]. Due to the multifactorial nature of cancer, the likewise multi-targeting ability of curcumin makes it an attractive component for chemotherapeutic research.

Combinatorial treatment of cancer cells with curcumin and chemotherapeutic drugs indicate that curcumin increases the sensitivity of treated cells towards drug treatment and reduces the risks of drug resistance. Bordoloi and colleagues reported that the chemosensitizing effects of curcumin are attributed to its multitargeting nature; to date, there are approximately 160 in vitro studies that demonstrate curcumin acting in synergy with a wide range of chemotherapeutic drugs, including gemcitabine, genistein, paclitaxel, cisplatin, mitomycin C, doxorubicin, and vinblastine, to modulate anti-tumorigenic effects in approximately 16 cancer cell lines, and 62 in vivo studies in various rat and mice animal models [13,18].

Furthermore, results from clinical trials indicate that curcumin has a good safety profile and exhibits high potential as a chemotherapeutic agent, even when administered at high doses [19]. However, despite its impressive therapeutic properties and safety index, the relatively poor bioavailability of curcumin has been highlighted as a critical issue to solve before curcumin can be considered as a therapeutic agent.

## 2. Bioavailability of Curcumin

Following oral administration, curcumin passes through the alimentary tract and is extensively metabolised by reduction [20], sulfation, and glucuronidation in the liver, kidney, and intestinal mucosa [21] and poorly absorbed from the gut resulting in equally low uptake and tissue distribution [22,23]. In plasma, 99% of curcumin is present as less active glucuronide and sulphate conjugate metabolites [21]. There is greater metabolic conjugation and reduction in the GI tracts of humans than in rat intestinal tissue [24], indicating that, ultimately, human clinical trials are a better judge of the pharmacokinetic ability of curcumin. Curcumin has also been shown to be rapidly eliminated from the body, with a large proportion being excreted in faeces and its glucuronide and sulphate metabolites in urine. The half-life of curcumin in rats was reported as 28.1 ± 5.6h and 44.5 ± 7.5h after intravenous and oral administration, respectively [25].

In an effort to enhance curcumin bioavailability, the release of curcumin has been studied in nanoemulsions; in complexation with bioactive conjugates; in concomitant administration with natural products such as piperines, or complexed with micelles and phospholipid complexes; and encapsulated with PLGA, cyclodextrine and propoylene glycol, and liposomal carriers [13,15]. Each method has variable degrees of success in improving curcumin bioavailability by increasing its solubility, or increasing cellular uptake, absorption, tissue distribution, and curcumin half-life. However, the use of natural and synthetic analogues of curcumin as an option to overcome the limitations of curcumin but retain its safety and efficacy is also being considered.

## 3. Natural Analogues of Curcumin

The chemistry of curcumin in relation to its function is widely studied, because its reactivity is contributed by the conjugated alkene, phenol, and diketone moieties. Commercially available preparations of curcumin consists of 77% curcumin, 17% demethoxycurcumin (DMC), and 3% bisdemethoxycurcumin (BDMC), all of which structurally differ in the substitution on the aromatic ring [26]. These curcuminoids also display some degree of improved stability over curcumin. Comparative in vivo studies after intraperitoneal administration in mice revealed that curcumin and DMC were rapidly degraded, but that the latter was more stable [27]. Furthermore, enzymatic hydrolysis studies showed that BDMC is chemically more stable than curcumin and DMC [28].

Bioactivity studies show that DMC and BDMC activity on cancer cells are largely more reactive than curcumin. BDMC has been shown to have more potent anti-mutagenic and anti-carcinogenic activity compared to curcumin and DMC [29]. DMC, meanwhile, is more effective than curcumin and BDMC in inhibiting the proliferation of breast cancer cells [30]. BDMC and DMC also showed higher anti-metastatic properties than curcumin in inhibition of cancer cell invasion by significant downregulation of MMP-2 and MMP-9 enzymes involved in cleavage of the extracellular matrix [31]. DMC and BDMC, however, may exhibit cell-specific effects as treatment selectively up-regulated MMP-3 expression in MDA-MB-231 invasive breast carcinoma cells but not in MCF-7 non-invasive breast cancer cells [32].

Structure–activity relationship studies determined that the phenolic group and the 1, 7-diaryl-3, 5-dioxo-1, 6-heptadiene skeleton of curcumin are largely responsible for its anti-inflammatory and anti-oxidative properties. It is these components, therefore, that have been modified to study improvements in its activity. An account of various curcumin formulations, derivatives, and their roles in anticancer activity has been reviewed [33]. Interestingly, a majority of compounds with these structural alterations led to improved growth inhibitory activity over curcumin directed at similar anticancer molecular targets. FLLL32 [(2E,2’E)-1,1’-(cyclohexane-1,1-diyl)bis(3-(3,4-dimethoxyphenyl)prop-2-en-1-one] was designed to preferentially interact with critical domains of JAK2 and STAT3 and demonstrated greater inhibition of STAT3 phosphorylation and its downstream targets in human rhabodomysarcoma cells, human multiple myeloma, glioblastoma, liver cancer, breast cancer, pancreatic cancer, and colorectal cancer cell lines over curcumin [34,35,36]. It has been reported that treatment of cisplatin-resistant head and neck squamous cell cancer (HNSCC) cells with FLLL32 is able to induce apoptotic cell death by sensitizing the cells to lower doses of cisplatin [37].

HMBME (4-hydroxy-3-methoxybenzoic acid methyl ester) was developed to target the Akt/NF-κB signaling pathway and was able to inhibit the proliferation of human and mouse PCA cells by reducing phosphorylated Akt and its kinase activity, transcriptional activity of NF-κB and its DNA binding activity, as well as p65 expression. The data indicated that HMBME-mediated inhibition of Akt kinase activity has the potential to suppress survival or anti-apoptotic pathways [38]. Difluoronated curcumin (CDF) downregulated NF-κB in MIAPaCa-2 pancreatic cells and showed selective accumulation in the pancreas, making it a potential chemotherapeutic agent for pancreatic cancers. CDF also caused inhibition of AR/TMPRSS2-ERG/Wnt signalling and prostate cancer cell invasion [39].

## 4. Diarylpentanoids as Potential Therapeutic Curcumin Analogues Treatment of Cancer Cells

The major problem highlighted by the clinical use of curcumin is its poor aqueous solubility, low bioavailability, and intense staining colour. Despite its limitations, curcumin remains as an ideal lead compound for the design of more effective analogues. Curcumin analogues denote all compounds that are structurally analogous to curcumin. By making specific substitutions at the aromatic rings and heterocyclic linker, analogues can exhibit enhanced therapeutic efficacy. It was reported that the 3-oxo-1,4-pentadiene linker of curcumin was the basis for its potent cytotoxic effect.

Diarylpentanoids (DAPs) or 1,5-diaryl-3-oxo-1,4-pentadienes are curcumin analogues that displayed greater growth-suppressive activity over curcumin and other diarylheptanoids (7-carbon curcuminoids). DAPs contain a five-carbon space between its aryl rings and feature the basic component of an “improved curcumin”. The evidence for the anticancer therapeutic potential of diarylpentanoids is found in the results of numerous studies that have reported the growth-inhibitory and apoptosis-inducing potential of DAPs in a wide range of cancer cells.

The evidence for the anticancer therapeutic potential of diarylpentanoids is found in the results of numerous studies that show that diarylpentanoids induce growth-suppressive effects in a wide range of cancer cell lines. The compounds (in parentheses) exerted growth inhibitory effects in colorectal (GO-Y035, GO-Y030, FLLL-11, FLLL-12, HO-3867, and EF24), breast (GO-Y035, GO-Y030, HO-3867, EF24, and EF31), pancreatic (GO-Y035, GO-Y030, FLLL-11, FLLL-12, and EF31), prostate (HO-3867, EF24, and ca27) [40], lung (GO-Y035, HO-3867, and EF24), thyroid gland (GO-Y035 and GO-Y030), liver bile duct (GO-Y030), ovarian (HO-3867, EF24, and EF31), stomach (GO-Y035), and cervical cancers (EF24). In most of these cases, many diarylpentanoids were able to induce effects in more than one cancer type and exhibited levels of inhibition that were several fold higher than curcumin.

As opposed to the vast amount of literature that discusses clinical and preclinical studies involving curcumin, the research for diarylheptanoids and diarylpentanoids is currently ongoing. However, judging from the improved antitumor activity of diarylpentanoid analogues, it is reasonable to propose that these compounds have significant chemotherapeutic potential over curcumin. Recent docking studies have suggested that they may be used as lead compounds to further design cytostatic agents with improved anti-cancer effects [41]. Furthermore, since bis(arylmethylidene)acetones have been shown previously to be strong Michael acceptors [5] it has been postulated that these compounds may interact with key target molecules in order to induce its action.

## 5. Molecular Mechanisms of Diarylpentanoids: Effect on Molecular Pathways

**Figure 2:**
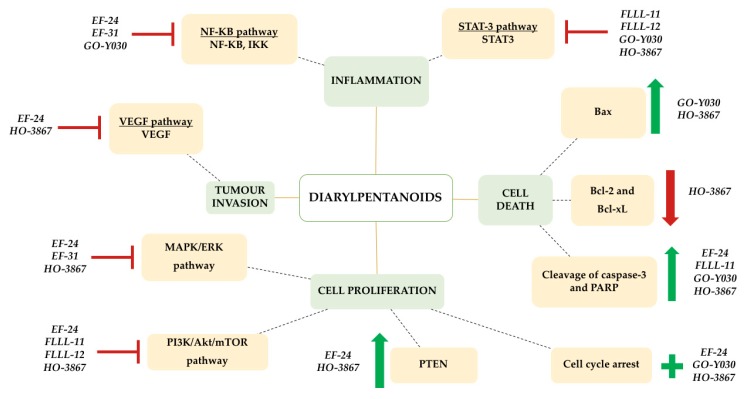
The effects of diarylpentanoids (DAPs) on inflammation, cell death, cell proliferation, and tumour invasion in in vitro and in vivo cancer models by the modulated expression of specific target molecules/pathways (yellow boxes). Green arrows denote up-regulated expression and red arrows denote down-regulated expression. Pathways that have been inhibited by particular DAPs are expressed using a red ‘
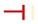
’ symbol, while induction is represented by a green ‘**+**’ symbol.

Molecular targets of selected DAPs that demonstrate anticancer effects (Table 1) are therefore a critical area of study to further elucidate its mechanism of antitumor action and chemotherapeutic potential. This review paper focuses on selected DAPs that have been studied for its molecular mechanism with identified molecular targets that exert anticancer activity. Figure 2 encapsulates the potential effects of diarylpentanoids on key target molecules and signalling pathways. 

### 5.1. Effects on NF-κB Signalling Pathway

The NF-κB signalling pathway is made of up of a group of closely related protein dimers that are sequestered in the cytosol of unstimulated cells by inhibitors known as IκB proteins. Once the canonical pathway is triggered, the activation of Iκβ kinase complex, known as IKK, phosphorylates the inhibitors binding NF-κB. The action results in targeting the inhibitors for ubiquitin-dependent degradation, thus liberating NF-κB dimers and allowing for their translocation to the nucleus where they modulate various transcriptional functions. The target genes of NF-κB are involved in inflammation, anti-apoptosis, and regulation of cell proliferation, all of which can contribute to tumorigenesis [57]. They have been shown to stimulate transcription of G1 cyclin genes [58] and to confer resistance to cancer therapies by diminishing the apoptotic response [59] by increasing metastasis.

Diarylpentanoids have been shown have an effect on the various components of the NF-κB pathway, specifically by causing inhibition in its activity and phosphorylation. Kudo et al. found that GO-Y030 decreased TNF-alpha-induced NF-κβ transactivation to a degree that was more potent than even curcumin [43]. The same compound was also found to have an effect on the inhibitors and kinase complex. The amount of phosphorylated Iκβα decreased after treatment along with the expression of other target genes that confer malignancy, such as COX-2, c-Myc, cyclin D1, and XIAP [47]. EF24, a diarylpentanoid that has been eliciting a wide amount of interest in its anticancer properties, has been shown to potently suppress the NF-κB signalling pathway by direct action on IKK by inhibiting the phosphorylation of its substrate, Iκβ, subsequently causing IKK to be degraded [54].

EF31, which is structurally related to EF24, was shown to exhibit significantly lower phosphorylation of NF-κB p65 protein [44] and greater inhibition of IKKβ compared to either EF24 or curcumin. It exhibits both anti-inflammatory and anticancer activity in NF-κB -dependent cancer cell lines while demonstrating minimal toxicity in RAW macrophages [56]. Similar to EF24 action, EF31 interacts with IKKβ and blocks phosphorylation of Iκβ necessary for NF-κB activation and nuclear translocation. As yet, the molecular mechanism by which this occurs is unknown, but a proposed mechanism suggests that it may be structural in nature; EF31 may partially bind to the ATP binding site of IKKβ, thus blocking the phosphorylation of Iκβ. Studies performed with related enones also exhibit inhibition of TNFα-induced activation of NF-κB that may occur at the level of the kinase complex [9,60].

### 5.2. Effects on STAT3 Signalling Pathway

Signal transducers and activators of transcription proteins (STATs) are cytoplasmic transcription factors that are only activated in cases of specific receptor stimulation by activation of Janus kinases (JAKs). STAT dimers are translocated into the nucleus and once there, STAT dimers bind to specific sequences to activate or repress transcription of target genes. (For reviews see [60,61]). Several studies have validated that increased activity of STAT3 contributes to tumorigenesis by enhancing tumour growth and invasion [62].

Curcumin has been shown to inhibit STAT expression, specifically regarding the activity of STAT3, and most of the studies performed to investigate the effect of diarylpentanoids on tumours focus on STAT3 over STAT1 and STAT2. There appears to be selectivity with respect to the effect of diarylpentanoid treatment on the STAT proteins, as discovered by one such study that showed that treatment with GO-Y030 has been shown to inhibit STAT3 phosphorylation in colorectal cells with selectively higher inhibition of STAT3 over STAT1 and STAT2 [46]. By comparison with the parent compound, curcumin, inhibition of STAT3 was also found to occur significantly in breast and pancreatic carcinoma cells at concentrations at which even higher concentrations of curcumin were unable to induce an effect [45].

The inhibition was confirmed both in this study and in another investigation after treatment with FLLL-11 and FLLL-12 [48]. The Friedman group analysed the inhibition of phosphorylation on the Tyr705 residues of STAT3, the results of which were mirrored after HO-3867 treatment. Here, treatment with HO-3867 induced expression of classic apoptotic markers of cleaved caspase-3 and PARP, which were shown to be facilitated by the inhibition of STAT3 phosphorylation at Tyr705 and Ser727 residues [49]. 

Another interesting finding was that treatment with HO-3867 induced potential cytotoxicity in cancer cells but not in non-cancerous cells. This differential cytotoxicity mediated through inhibition of STAT3 activation in cancer cells while providing antioxidant protection to healthy cells suggests that antioxidant-conjugated diarylpentanoids would be useful as safe anticancer agents for cancer therapy [49]. HO-3867 treatment has also been shown to significantly reduce JAK1 phosphorylation and reduction in STAT3 downstream target protein levels including Bcl-xL, Bcl-2, and vascular endothelial growth factor, suggesting that exposure to HO-3867 interrupted the JAK/STAT signalling pathway [50].

### 5.3. Effects on Akt Phosphorylation and PTEN Expression

The PI3K/PTEN/Akt/mTOR signalling pathway is a major focus of attention due to its critical role in many cellular processes, specifically in that of cancer growth, survival, and motility. The signalling pathway, named after its major members, is activated by growth factor or cytokine receptor ligation. Activated PI3K causes downstream phosphorylation of Akt, which is an important cell growth regulator. Dephosphorylated Akt has been shown to have apoptotic properties [63], but nuclear-activated Akt may be antiapoptotic in nature [64,65]. Increased PI3K signalling is often due to mutational alteration in members of the pathway [as summarised by [66]] or loss of the tumour suppressor, PTEN, which acts to dephosphorylate a product of PI3K and inhibits the signalling pathway.

In terms of the PI3K/PTEN/Akt/mTOR pathway as a molecular target, FLLL-11 and FLLL-12 diarylpentanoids have been shown to inhibit Akt phosphorylation at half the dose at which curcumin is required to achieve the same result [48]. The effects of EF24 on Akt were observed in the significant dose- and time-dependent decrease in Akt phosphorylation in treated cells [52].

In another, EF24 has been shown to upregulate phosphorylated PTEN expression through the inhibition of ubiquitin-mediated degradation. In fact, suppression of PTEN expression with siRNA significantly reduced p53 and p21 and activated Akt phosphorylation, leading to increased cancer cell survival, while overexpression of PTEN markedly induced apoptosis [53]. The authors postulate that upregulation of PTEN inhibited Akt and MDM2, which enhanced p53 expression and induced G2-M arrest and apoptosis. Immunofluorescence studies also showed that PTEN expression and activation was induced as early as one hour after treatment with HO-3867, confirmed by RT-PCR, in an increase of PTEN mRNA expression in three hours. ATF2, the transcription factor of PTEN, was also activated within 15 min of treatment [67]. In order to confirm that PTEN was causing the induction of G1 cell cycle arrest, the group treated PTEN siRNA-transfected smooth muscle cells with HO-3867 and found repression in PTEN expression. They thereby concluded that HO-3867 increased PTEN expression by activation of ATF-2 binding to the PTEN promoter.

### 5.4. Effects on MAPK/ERK Signalling Pathway

The Ras/Raf/MEK/ERK (or MAPK/ERK) pathway is critical for cell proliferation and its signalling components are mitogen-activated protein kinases (MAPKs). Binding of an extracellular mitogen to a membrane ligand causes activation of Ras kinases, which in turn activates Raf kinases. Raf kinases are required to phosphorylate mitogen-associated/extracellular-regulated kinase-1, MEK, which subsequently activates extracellular regulated kinases, ERK1 and ERK2 [65]. Phosphorylated ERK proteins can activate a variety of substrates, specifically transcription factors involved in stimulating growth and proliferation. Two other major MAPK pathways are the Jun N-terminal kinase (JNK) and p38 MAPK pathways; these are mainly activated by genotoxic stresses and play major roles in controlling cell proliferation, differentiation, and survival. Cancers have been known to disrupt these pathways in their favour, but they have been shown to have tumour suppressive function in other cell types [68].

Interestingly, it was found that EF24 triggered a negative feedback loop through p38 activation and that a combination of EF24 and a p38 inhibitor produced a synergistic inhibition of proliferating activity of A549 lung cancer cells and induced their apoptosis. Silencing of p38 sensitized the lung cancer cells to EF24-induced death. Inhibition of ERK and JNK, however, did not significantly affect EF24-induced cytotoxicity [55]. EF31 was observed to inhibit MAPK pathways and significantly inhibited the DNA-binding activity of transcription factors AP1, ATF-2, and c-JUN [56].

Several studies investigated links between both PI3K/Akt and MAPK/ERK pathways. An investigation involving treatment with HO-3867 found that it led to activation of PTEN-inhibited G1 cell cycle arrest by induction of p53 and p21 and downregulation of ERK1/2 [67]. Treatment with EF24 found a concurrent decrease in AKT phosphorylation as well ERK phosphorylation in treated cells. Wei et al. (2012) reported one diarylpentanoid compound (E10) that showed a strong decrease in the level of phosphorylated Akt as well as in EGF-mediated ERK phosphorylation, while curcumin showed little or no inhibition [69].

### 5.5. Effects on VEGF Signaling Pathway

Vascular endothelial growth factor (VEGF) induces vasculogenesis and is a critical regulator of angiogenesis, a feature crucial for tumorigenesis. Treatment with HO-3867 was shown to significantly reduce VEGF, which is a downstream target of the JAK/STAT3 signalling pathway. EF24 treatment was also shown to suppress constitutive H_2_O_2_-induced VEGF expression in ovarian cancer cells at a post-transcriptional level [51].

### 5.6. Induction of Cell Cycle Arrest and Apoptosis Pathways

Caspase activation executes a process of programmed cell death that induces DNA fragmentation and morphologic changes [70], which include blebbing, cell shrinkage, nuclear fragmentation, and chromatin condensation [71]. Poly(ADP-ribose) polymerases (PARPs), on the other hand, are nuclear protein enzymes that facilitate DNA repair. Hence, chemotherapeutic treatments that activate effector caspases, such as caspase-3, -6, and -7, and upregulate PARP cleavage are worthwhile avenues to explore.

Most of the diarylpentanoids studied activate caspase-3 activation and cause PARP cleavage to a greater extent that curcumin. These examples include GO-Y030, FLLL-11, HO-3867, and EF24 [42,46,47,48,50,55,72]. Many of these diarylpentanoids also increase the expression of pro-apoptotic proteins. GO-Y030 and HO-3867 have been shown to increase the ratio of Bax:Bcl-2 and Bax:Bcl-xL ratios, suggesting that the cells were undergoing apoptosis [50,52].

The very definition of cancer indicates uncontrolled cell growth that overrides cell cycle checkpoints; thus, an invaluable target of chemotherapy is abrogation of the cell cycle that can lead to cell death. EF24 showed an increased number of cells in G2-M phase [52] and HO-3867-induced G2-M cell cycle arrest in A2780 ovarian cancer cells by modulating cell cycle regulatory molecules p53, p21, p27, cyclin-dependent kinase 2, and cyclin [50]. In fact, GO-Y030 showed G2-M arrest, even in the absence of inhibited caspase-3 and caspase-8 [48].

## 6. Concluding Remarks and Future Directions

Although curcumin is readily occurring, has a range of health benefits, and is a dietary requirement for many individuals, it is nonetheless difficult to be patented or considered as a therapeutic agent. It has, however, been suggested that its structure can be used as a lead to design a more effective compound, and analogues with five carbon spacers between its aryl rings have been shown to have improved cytotoxicity over the parent compound that possesses a longer carbon bridge as part of its structure. Of course, there is ample research that investigates the specifics of the structure–activity relationship of many synthetic curcumin analogues, and while it is important to study the components of the structure of the analogue that allow it to interact with key molecules, it is merely one segment of a much larger picture.

It is equally important to study which molecules are targets of these diarylpentanoids, as this knowledge can be used to research their potential therapeutic benefits. This knowledge would be even more crucial in the context of cancer; because it is a multifactorial disease that may involve many pathways, it is critical that chemotherapeutic drugs have the ability to activate more than one pathway. As observed in Table 1, diarylpentanoids target similar molecules and pathways and there is evidence that they also exert their antitumorigenic effects to a greater degree than curcumin.

In fact, the requirement for structural analogues arose from the need to improve upon curcumin potency and bioavailability but maintain its pharmacological safety and tumorigenic targets. Diarylpentanoids appear to fulfil the requirement for increased potency while maintaining similar tumorigenic targets, but it must be acknowledged that the research for increased bioavailability and safety needs to be strongly established using diverse animal models before proceeding with clinical trials. Further investigations also need to be performed to confirm potential drug interactions or combinations of treatments to explore the synergistic action of diarylpentanoids with currently approved treatments for an increased therapeutic response.

Even with respect to molecular targets, there is still much that needs to be explored. Most of the research appears to be focused on assessing the chemotherapeutic effect of the diarylpentanoids on isolated targets, but genome-wide assays may prove to be more useful in elucidating other genes or identifying novel pathways involved in cancer.

Hence, the knowledge of the intracellular targeting of diarylpentanoids in cancer is steadily increasing but this area needs to be developed in tandem with the information on its bioavailability and safety in order to form a holistic perspective of the potential efficacy of diarylpentanoids in chemotherapy.

## Figures and Tables

**Table 1:** Proposed molecular pathways modulated by diarylpentanoids (DAPs) in vivo and in vitro.

DAP	Human Cancer Cell Line (In Vitro)	Source	In Vivo	Proposed Modulated Molecular Pathways	Reference
**GO-Y030**	SW480 HT29 HCT 116	Colorectal Colorectal Colorectal	-	NF-κB, STAT3, Cell cycle arrest and apoptotic pathways	[42]
	HCT116	Colorectal	-		[43]
	HCT116	Colorectal	-		[44]
	MDA-MB-231	Breast	-		[45]
	ALDH+/CD133+ stem cells from cell lines SW480, HCT-116, DLD-1 and HT29	Colorectal Cancer stem cells	-		[46]
	SW620 SH-10-TC MCF7 PC3 PK-1 8505c HuCCT-1	Colon Stomach Breast Prostate Pancreas Thyroid Bile duct	-		[47]
**FLLL-11**	SW480 HT29 HCT 116	Colorectal Colorectal Colorectal	-	STAT3, PI3K/PTEN/Akt/mTOR, cell cycle arrest and apoptotic pathways	[42]
	PANC-1 BXPC-3 MIA-PACA-2 ASPC-1 HPAC	Pancreas Pancreas Pancreas Pancreas Pancreas	-		[48]
**FLLL-12**	SW480 HT29 HCT 116	Colorectal Colorectal Colorectal	-	STAT3, PI3K/PTEN/Akt/mTOR, cell cycle arrest and apoptotic pathways	[42]
	PANC-1 BXPC-3 MIA-PACA-2 ASPC-1 HPAC	Pancreas Pancreas Pancreas Pancreas Pancreas	-		[48]
**HO-3867**	A2780 A2780R MCF7 HCT116 PC3 HepG2 A549 SCC4	Ovarian Ovarian Breast Colorectal Prostate Liver Lung Squamous Cell	-	STAT3, PI3K/PTEN/Akt/mTOR, MAPK/ERK pathway VEGF signalling, cell cycle arrest and apoptotic pathways	[49]
	A2780 SKOV3 OV4 OVCAR3	Ovarian Ovarian Ovarian Ovarian	Human ovarian xenograft (A2780) grown in back of BALB/C nude mice		[50]
**EF24**	IGROV1 SK-OV-3	Ovarian Ovarian	-	NF-κB, PI3K/PTEN/Akt/mTOR, MAPK/ERK pathway, VEGF signalling, cell cycle arrest and apoptotic pathways	[51]
	HCT-116 HT-29 AGS	Colorectal Colorectal Stomach	HCT-116 colon cancer xenografts established in athymic nude mice		[52]
	A2780R	Ovarian	-		[53]
	A549, H460 Calu-1 1A9 MDA-MB-231 HeLa	Lung Lung Ovarian Breast Cervical	-		[54]
	A549	Lung	-		[55]
**EF31**	A2780 MDA-MB-231	Ovarian Breast	-	NF-κB, MAPK/ERK pathway	[56]
			Human head and neck squamous cell carcinoma Tu212 xenograft tumors established in athymic nude mice		[44]

Abbreviations: activating transcription factor 2 (AFT2), extracellular signal-regulated kinase (ERK), I kappa B kinases (IKK), c-Jun NH2-terminal kinase (JNK), Mitogen-activated protein kinase (MAPK), nuclear factor kappa-beta (NF-κB), Phosphatase and tensin homolog (PTEN), signal transducer and activator of transcription (STAT), vascular endothelial growth factor (VEGF).
